# Deep Brain Stimulation—Possible Treatment Strategy for Pathologically Altered Body Weight?

**DOI:** 10.3390/brainsci8010019

**Published:** 2018-01-22

**Authors:** Philip Prinz, Andreas Stengel

**Affiliations:** 1 Department for Psychosomatic Medicine, Charité Center for Internal Medicine and Dermatology, Charité-Universitätsmedizin Berlin, Corporate Member of Freie Universität Berlin, Humboldt-Universität zu Berlin, and Berlin Institute of Health, 12200 Berlin, Germany; philip.prinz@charite.de; 2 Department of Psychosomatic Medicine and Psychotherapy, Medical University Hospital Tübingen, 72076 Tübingen, Germany

**Keywords:** anorexia nervosa, binge-eating disorder, depression, obesity, Parkinson’s disease

## Abstract

The treatment of obesity and eating disorders such as binge-eating disorder or anorexia nervosa is challenging. Besides lifestyle changes and pharmacological options, bariatric surgery represents a well-established and effective-albeit invasive-treatment of obesity, whereas for binge-eating disorder and anorexia nervosa mostly psychotherapy options exist. Deep brain stimulation (DBS), a method that influences the neuronal network, is by now known for its safe and effective applicability in patients with Parkinson’s disease. However, the use does not seem to be restricted to these patients. Recent preclinical and first clinical evidence points towards the use of DBS in patients with obesity and eating disorders as well. Depending on the targeted area in the brain, DBS can either inhibit food intake and body weight or stimulate energy intake and subsequently body weight. The current review focuses on preclinical and clinical evidence of DBS to modulate food intake and body weight and highlight the different brain areas targeted, stimulation protocols applied and downstream signaling modulated. Lastly, this review will also critically discuss potential safety issues and gaps in knowledge to promote further studies.

## 1. Introduction

More than 35% of men and women are overweight or obese [1]. Overweight is defined by a body mass index (BMI; kg/m^2^) between 25–30 kg/m^2^, whereas obese subjects have a BMI of more than 30 kg/m^2^. Unfortunately, the prevalence of obesity does not only increase in adults but also in children and adolescents [1]. It is expected that the prevalence of obesity will continue to rise and with that obesity-associated diseases, including insulin resistance, type 2 diabetes, coronary heart disease or stroke, malignomas and psychiatric comorbidities [2,3,4]. Current treatment strategies encompass changes in lifestyle including dietary approaches and increased physical activity as well as psychotherapy [5]. Pharmacological treatment plays a minor role so far; while few drugs are on the market, including Orlistat (prevents lipid absorption), Liraglutide [a glucagon-like peptid (GLP)-1 analogue] or Lorcaserin (a subtype selective serotonin receptor agonist), others are in the pipeline such as Metreleptin (a leptin analogue), Pramlintide (an amylin analogue) or Topiramate [a gamma-aminobutyric-acid (GABA) inhibitor] [6]. Nonetheless, it is to note that pharmacological treatment only induces a moderate effect (~6% excess weight loss) [7]. The most effective, although invasive, treatment option is bariatric surgery inducing a reduction of ~20% excess weight loss in five years [7]. Binge-eating disorder (BED) is often associated with obesity and reflected by the consumption of large quantities of food in a short period of time and the loss of control [8]. Moreover, it is accompanied by negative emotions such as guilt and shame afterwards [8]. The prevalence of BED is 1–3.5% in the general population and up to 15% in adolescents and young adults [8]. BED patients undergo behavioral therapy often in combination with medication such as selective serotonin reuptake inhibitors or anti-obesity drugs (including Lorcaserin) [9].

Anorexia nervosa (AN) is an eating disorder reflected by the patients’ desire to lose body weight or to maintain body weight at a lower level than adequate for their corresponding age and height [10]. AN patients suffer from body image disturbance and have an extreme fear to regain body weight [11]. The incidence of AN is at least eight people per hundred thousand per year, with an overall prevalence of 0.3% [12] and a peak in young women and adolescent girls [13]. The pathogenesis of AN is still incompletely understood and includes genetic, neuronal, social and environmental factors [14]. Treatment options encompass structured care and psychotherapy, whereas specific pharmacological treatment options are still missing [12,14].

Deep brain stimulation (DBS), an implantation of intracranial electrodes, was first described by the Spanish neuroscientist José M. Delgado in the early fifties of the 20th century [15]. He showed that electric stimulation of various areas of the brain in cats or monkeys exerts effects on the animals’ behavior and motor activity [15]. In 1963, the Russian neuroscientist Natalia P. Bekthereva was the first to demonstrate the use of chronic DBS of subcortical structures for the treatment of motor disorders [16], followed by first experiments in psychiatric disorders performed by the Norwegian neurophysiologist and psychiatrist Carl W. Sem-Jacobsen [17]. He safely implanted electrodes in the thalamus to describe adequate target sites in the treatment of Parkinson’s disease (PD) [17]. Subsequently, more research was performed on DBS, investigating its effects on chronic pain [18], vigil coma [19] or epilepsy [20].

Today, DBS is considered safe and well tolerated in the treatment of PD [21]. Especially the subthalamic nucleus (STN) is an interesting target for DBS, resulting in an improvement of wellbeing [22], motor symptoms [23] and gait function [24] in patients with PD. Since DBS is known to influence the neuronal network, it is also tested in various other diseases, including Tourette’s syndrome [25,26], epilepsy [27,28], depression [29,30] or in the treatment of obesity and eating disorders [31,32,33]. Since the treatment of obesity and eating disorders is challenging as described above, new approaches are desired to modulate food intake and body weight. Therefore, the application of DBS might be a promising approach to influence the neuronal network of patients with obesity and eating disorders.

Several studies investigated the effects of DBS on body weight gain and food intake in rats [34,35,36,37,38], pigs [39], monkeys [40] and humans [31,32,33,41] under different conditions of chronically altered body weight (obesity and AN). Since various areas of the brain are known to be involved in the modulation of food intake [42], DBS might target different brain sites including the subcallosal cingulate (SCC) [33,41], the reward circuitry [34,35,36], internal globus pallidus (GPi) [43], hypothalamic areas [31,39,40], STN [44,45] or the rostromedial tegmental nucleus (RMTg) [46].

The aim of this review was to summarize the preclinical and clinical evidence on the potential use of DBS in the treatment of obesity and eating disorders, namely BED and AN. Therefore, we will discuss the effects of DBS on food intake and body weight in animal studies and will describe studies that performed DBS in patients with obesity or eating disorders as well as other diseases that might pave the way for a prospective application of DBS in the treatment of eating disorders. Moreover, the underlying mechanisms will be mentioned. Lastly, hurdles and gaps in knowledge will be highlighted to promote further research in this lively field.

## 2. Effects of DBS on Food Intake and Body Weight Gain

It was shown that the medial shell of the nucleus accumbens (NAcc) as part of the reward circuitry is involved in the regulation of food intake [47]. The authors showed that pharmacological bilateral inhibition of alpha-amino-3-hydroxy-5-methylisoxazole-4-propionic acid (AMPA) and kainate glutamate receptors in the medial shell increased food intake in rats [47]. This discovery indicated the medial shell of the NAcc as an interesting target for the application of DBS to modulate food intake and subsequently body weight gain. In 2012, a study investigated the effects of bilateral DBS of the core, lateral and medial shell of the NAcc, using biphasic stimulation, a frequency of 130 Hz and different electric currents from 0 to 100 µA [36]. The study showed that none of the used stimulation parameters affected food intake if applied in the NAcc core or lateral shell. In the medial shell, however, DBS with 100 µA increased food intake one hour after the start of the stimulation, whereas all other parameters did not affect food intake [36]. Although body weight was not assessed, this study paved the way for further research using the medial shell of the NAcc as a potential target site to influence food intake and body weight in rodents. Subsequent studies by our group confirmed the food intake-stimulatory effect in the very first hour after DBS in rats using the same stimulation parameters (biphasic, 130 Hz, 100 µA) [34]. Moreover, continuous DBS of the medial shell of the NAcc moderately increased body weight in rats after seven days compared to sham stimulation without affecting overall food intake [34]. Interestingly, a circadian shift of food intake was observed with an increase of food intake during the light phase and a reduction of food intake during the dark phase without alterations in 24-hour food consumption [34]. These results show that DBS of the NAcc medial shell exerts a rapid onset effect on food intake (as reflected by the orexigenic effect during the first hour following stimulation [34,36]) without affecting overall food intake (as reflected by unaltered food consumption over 7 days [34]). However, the shift of food intake to the light phase (the phase where rats usually do not eat [48]), might contribute to the observed increase in body weight gain and therefore indicates the NAcc as interesting target site when a stimulation of food intake and body weight is desired (e.g., in AN). Since no alterations in behavior were detected, these results might also point towards an impact of DBS on energy expenditure. Another study did not detect any impact of DBS of the medial shell of the NAcc on body weight gain in normal weight rats using different stimulation parameters (130 Hz, 500 µA) [35], again showing the importance of the DBS parameters used.

It is to note that these experiments were performed in normal weight rats. However, several signaling pathways of food intake regulation are altered under conditions of obesity [49]. Therefore, one might assume that DBS of the NAcc shell has different effects under conditions of obesity. In line with this assumption, a study showed that DBS of the NAcc shell (130 Hz, 500 µA) over 14 days decreased daily caloric intake and body weight in diet-induced obese (DIO) rats, whereas food intake in normal weight rats was not altered [35]. Similarly, continuous DBS in DIO mice for four days (160 Hz, 150 µA) decreased caloric intake and subsequently body weight [50]. The authors showed that extracellular dopamine levels and the mRNA expression of the dopamine D2 receptor were increased in the NAcc shell of DBS DIO rats compared to sham-treated DIO rats [35]. Since dopamine D2 receptor availability was decreased in several brain regions (e.g., amygdala and hypothalamus) in obese subjects undergoing bariatric surgery [51], a modulation of central dopamine signaling might well be involved in the reduction of food intake and subsequently body weight. Whether species differences (rat/mouse vs. human), site-specific effects (NAcc vs. amygdala or hypothalamus) or the different methods (DBS vs. bariatric surgery) account for the differential effects on dopamine signaling (increase vs. decrease) will have to be further investigated. The underlying mechanisms remain to be unraveled but these results might give rise to the possible use of DBS of the medial shell of the NAcc in the treatment of obesity. However, the results shown here must be interpreted with caution and subsequent studies focusing on the NAcc shell are desired. Nonetheless, DBS of the NAcc shell is a promising approach to influence food intake and body weight as shown in animal studies. Interestingly, DBS of the NAcc shell has differential effects under normal weight and obese conditions with a reduction of food intake under conditions of obesity by activation of the central NAcc dopamine system, whereas in normal weight rats light phase food intake is increased.

The application of DBS of the NAcc shell was also investigated in a mouse model of binge eating [50]. Binge eating was defined as more than 25% of a mouse’s daily caloric intake within an one-hour period with access to a high fat food pellet. Acute DBS (160 Hz, 150 µA) during this binge-eating period significantly reduced the high fat diet intake [50]. Interestingly, only DBS of the NAcc shell but not of the NAcc core or the dorsal striatum (as part of the reward circuitry) affected binge-eating behavior [50]. Activation of neuronal activity in the NAcc shell by DBS was shown using c-Fos immunoreactivity [50]. Furthermore, the DBS-induced reduction of high fat diet consumption was blunted by pretreatment with raclopride (a dopamine D2 receptor antagonist) [50]. A subsequent study investigated the effects of bilateral DBS of the NAcc core in binge-eating rats. Binge eating was induced by access to sweet fat diet pellets, which were provided daily for two hours in addition to regular chow and water. Intake of the highly palatable food, regular chow and water was measured for two hours [52]. DBS (150 Hz, 150 µA) reduced binge-eating size during this two-hour period [52]. Since no effects of NAcc core DBS were observed in the BED mouse model [50], one could speculate about species differences. Further research is needed to elucidate the role of the NAcc as potential target for DBS in BED models.

The hypothalamus is a brain area containing several nuclei involved in the regulation of food intake [42], including the paraventricular nucleus (PVN), the lateral hypothalamic area (LHA), the arcuate nucleus (Arc) and the ventromedial hypothalamus (VMH). Therefore, several studies were conducted investigating the effects of DBS targeting nuclei of the hypothalamus. Continuous bilateral DBS of the LHA in rats fed a high fat diet (180–200 Hz, 2 V) did not alter food intake, but significantly reduced body weight over a period of 24 days [53]. Subsequent research investigated the effects of bilateral DBS of the LHA of obese Zucker rats, which was performed for one hour per day for 15 consecutive days with 130 Hz and 300 µA [37]. Interestingly, food intake was not altered during the first 10 days, but significantly reduced after 15 days in DBS-treated animals associated with a reduction in body weight [37]. Taken together, these results indicate a body weight-reducing effect of DBS of the LHA under conditions of obesity in rats. Whether the stimulation parameters or strain differences contribute to these different results will have to be further investigated.

In the VMH, unilateral DBS (10 µA) resulted in decreased body weight gain compared to sham-treated rats at high frequencies of 150 or 500 Hz [38]. Since food intake was not altered, the authors measured CO_2_ production in a respirometer. DBS of the VMH caused an increased CO_2_ production compared to sham treatment during the 1.5-hour stimulation period indicative of increased basal metabolism [38]. The effect of DBS of the VMH on body weight was confirmed by a subsequent study in female Göttingen minipigs that underwent bilateral DBS of the VMH (50 Hz, 0.5–1.5 mA) [39]. Minipigs were maintained on a restricted food regimen for one month after DBS surgery without stimulation. This period was followed by a two-months period with twice the amount of food to produce obesity. During this period the animals were stimulated. Interestingly, the VMH-stimulated animals gained significantly less body weight compared to the non-stimulated group [39], indicating that DBS of the VMH is also effective to reduce body weight under conditions of developing obesity. These results are further supported by the effects of DBS of the VMH in adult Macaca fascicularis monkeys. Three of four animals lost body weight (~8%) following eight weeks of unilateral DBS (monopolar, 80 Hz, 2 V), an effect accompanied by significant loss of body fat (17–19%) in all four animals [54]. Animals received a standard re-feeding meal for one hour which was significantly reduced in all animals undergoing DBS (80 Hz) compared to a non-stimulation control or stimulation frequencies of 30, 50 and 130 Hz, respectively [54]. This acute trial was followed by a chronic experiment that showed an increase of food intake in the washout period (DBS off) over three weeks following eight weeks of DBS (80 Hz, 2 V, monopolar) [54]. Similarly, DBS of the VMH (100 µA, 1 h every 12 h on 3 consecutive days) decreased food intake in dogs [55]. These results show that findings differ based on the hypothalamic nucleus targeted, the laterality of application (bilateral vs. unilateral) and the stimulation parameters. Additionally, current data point to species differences with no effects of DBS of the VMH in rats, controversial effects in monkeys and a reduction of food intake in dogs.

Another interesting target to reduce the desire to eat is the central nucleus of the amygdala. In rats, DBS of the rat central nucleus of the amygdala (20 Hz or 130 Hz, 125 µA) decreased consumption of sucrose pellets within 30 min and resulted in aversion of an otherwise liked sucrose taste [56]. Furthermore, DBS of the central nucleus of the amygdala reduced the animals’ motivation to press the lever for sucrose pellets [56]. These results show that the central nucleus of the amygdala is an interesting target to lower food intake by reducing the motivation and liking of tasty foods in rats, effects desired in the treatment of obesity.

The RMTg is located in the midbrain and connected to dopaminergic and serotonergic pathways. A study investigated the effect of DBS of the RMTg on mood-related behaviors including anxiety and depressive behavior in rats. In this context, another food intake test was performed where rats were fasted for 24 h and received 60 g chow afterwards. Rats that received bilateral low frequency DBS (10 Hz, 20 µA) ate significantly less compared to the sham-stimulated group [46]. It is important to note that DBS of the RMTg did not alter behavior as assessed by the Skinner task, elevated zero maze or open field test [46]. This makes the RMTg an interesting target for DBS to selectively reduce food intake. However, this finding should be confirmed and further described in follow-up studies.

In addition, other brain areas were shown to increase body weight following DBS. The GPi was stimulated in patients with PD. Bilateral DBS of the GPi (stimulation parameters not indicated) slightly reduced BMI in patients with PD after one year of stimulation (−0.14 kg/m^2^, not significant), whereas in patients with dystonia DBS of GPi slightly increased BMI (+0.14 kg/m^2^, not significant) [57]. A subsequent study in 19 patients with PD showed that bilateral, monopolar stimulation of the GPi (130 Hz, 2.6 V) resulted in a significant increase of mean BMI (+0.66 kg/m^2^) [43]. However, these studies were performed in patients with PD and at this point it remains to be further investigated whether these results are transferable to other conditions such as eating disorders.

Besides the GPi, the STN is a very promising target in the treatment of PD [22]. In 30 patients suffering from PD, DBS of the STN (DBS parameters were not mentioned) resulted in a significantly increased body weight gain after one year (14.8 ± 9.8%), whereas 46.5% of patients already reported weight gain during the first three months [58]. Subsequent studies in patients with PD confirmed these results, showing a mean body weight increase [44,59] or a mean increase of BMI [57,59] following DBS of the STN. Interestingly, a recently published study did not detect any effect of DBS of the STN (with and without optimal drug therapy) on BMI following a two-year stimulation period in patients with early stage PD [45]. These discrepancies might be due to a fairly high BMI (29.7 kg/m^2^) at the baseline of this study (which is close to obesity) [45], whereas other studies enrolled PD patients with a baseline BMI of 21.6 kg/m^2^ [58] or 24.8 kg/m^2^ [57]. Moreover, the stage of PD might also influence the results (early stage PD vs. advanced stage PD). As already mentioned for the GPi, the results of DBS of the STN have to be followed up in animal models for eating disorders.

In summary, DBS of different brain areas alters body weight not only by stimulating food intake but also by affecting energy metabolism or energy expenditure, as shown in animal studies. Therefore, future studies focusing on body weight regulation should also focus on other parameters besides food intake, including energy expenditure, behavior, metabolic rate (including utilization of different macronutrients) as well as the motivation to eat to further characterize the effect underlying the body weight-modulating effects of DBS. All animal and human studies that investigated the effects of DBS in distinct brain nuclei on eating behavior and body weight are summarized in Table 1.

Since pre-clinical and first clinical studies already provided data that DBS affected food intake and body weight, the application of DBS is discussed as a potential treatment strategy in obesity and eating disorders such as BED and AN. However, it should be kept in mind that DBS can induce side effects and therefore patients should be selected very carefully. Additionally, it should be kept in mind that some studies listed here were performed in patients with other diseases such as PD and only indicate the potential effect of stimulation in certain brain areas without providing scientific evidence for the treatment of eating disorders. The following sections highlight current data on DBS as a potential treatment option in obesity, BED and AN.

## 3. Potential Application of DBS in Obesity

Under conditions of obesity, the NAcc shell is a promising target to reduce food intake and body weight. However, data are still limited. Acute and chronic DBS (160 Hz, 150 µA) of the NAcc shell reduced intake of a high fat diet and body weight gain in DIO mice [50]. No differences in behavior including locomotion, freezing, body turning and pivoting were observed [50]. Subsequent research of an independent group confirmed these results in DIO rats undergoing DBS (130 Hz, 500 µA) of the NAcc shell and reported reduced intake of high fat diet and a decrease of body weight [35]. Unfortunately, the authors did not monitor behavior during the stimulation period. Since these results are different from the ones observed under normal weight conditions with a lack of a body weight-decreasing effect [34,35,36], potential side effects of DBS of the NAcc shell should be investigated under obese conditions as well to further approach the possible application in humans. Two case reports investigated the effects of bilateral DBS of the NAcc in obese individuals showing a reduction of body weight. In the first study a 47-year old obese woman lost 44 kg in 10 months following bilateral DBS of the NAcc (185 Hz, 3.5 V) without any side effects detected [60]. The second study described a 19-year old obese woman who lost 13.3 kg in 14 months following bilateral DBS of the NAcc (130 Hz, 2 mA) without any reported side effects [61].

DBS of the LHA resulted in decreased body weight gain in rats [37,53] without any signs of pain, reduction in movement or other behavioral changes during the experiments [37]. In humans, two out of three obese patients lost body weight (−12.3% and 16.4%) following bilateral DBS of the LHA (185 Hz, 1–7 V) without reporting serious adverse effects during a mean follow-up of 35 months [31]. The authors also investigated serum levels of food intake-regulatory peptides such as insulin, insulin-like growth factor, ghrelin, leptin, neuropeptide Y (NPY), agouti-related peptide (AgRP) and peptide tyrosine tyrosine (PYY), which were not altered. [31]. These results point towards direct DBS-induced neuronal alterations rather than humoral effects that underlie the reduction of body weight, a hypothesis that needs further confirmation in animal studies or clinical trials. Some mild side effects were detected including nausea, anxiety, and sensations of “feeling too hot or flushed”, but these were only transient and lasted less than five minutes and were usually noted during programming changes of the stimulator [31]. Moreover, DBS of the LHA had no negative effect on psychological parameters including anxiety, tension, depression, cognitive dysfunction, emotional lability and guardedness as assessed by the Millon Behavioral Medicine Diagnostic tool. Taken together, although current data are sparse, targeting the LHA might hold therapeutic potential but needs further investigation. Especially prospective clinical trials are warranted to investigate the application of DBS of the LHA in bigger cohorts of obese patients.

Besides the LHA, several studies targeted the VMH. In rats, unilateral DBS of the VMH did not result in any abnormal behavior at frequencies of 150 or 500 Hz and 10 µA. Only higher currents of 40 µA resulted in freezing behavior at the onset of the stimulation [38]. Bilateral DBS of the VMH (50 Hz, 0.5–1.5 mA) in female Göttingen minipigs did not induce adverse effects on motoric and affective behavior between the pre-surgery and post-surgery period as observed by veterinaries blinded to the treatment. Additionally, no adverse reactions were observed during the initial activation of DBS [39]. These animal data might indicate safe applicability of DBS in the VMH. In humans, one study reported a rapid weight loss of 12 kg over five months without obvious changes in diet or habits in an obese patient [32]. It is important to note that side effects were not systematically reported in this case report. During the last four months, the patient switched off the stimulator at night because the subjective quality of sleep was better without stimulation [32]. The availability of one case report clearly indicates the need for larger randomized controlled studies further investigating the effects (and side effects) of DBS in the VMH of obese patients.

## 4. Potential Application of DBS in Binge-Eating Disorder

Since DBS is a promising approach in the treatment of obesity, it might also be used in the treatment of BED, often associated with or leading to obesity [8]. As already mentioned above, DBS of the NAcc shell (160 Hz, 150 µA) improved binge eating in mice [50]. Additionally, DBS of the NAcc shell tended to increase the latency to consume high fat food [50]. No differences in behavior were observed [50], possibly pointing towards a safe applicability of DBS in the NAcc shell in mice. It is to note that a study investigating the effects of DBS of the NAcc core (150 Hz, 150 µA) on binge eating in rats also showed reduction of binge-eating size without observing behavioral changes [52].

In humans, only one study investigated the effects of DBS of the LHA (bilateral, 185 Hz, 1–7 V) in three patients with BED. Binge eating improved in the patient reporting a severe binge-eating range preoperatively (as assessed using the Gormally Binge Eating Scale), while in the other two patients with moderate binge eating no effects were observed [31]. These findings point towards the need for further larger controlled studies that distinguish between severe and moderate BED when investigating the effects of DBS of the LHA.

Another study applied DBS in the VMH (bilateral, 50 Hz). A male patient with a BMI of 55.1 kg/m^2^ receiving DBS of the VMH reported reduced cravings and a decreased tendency to binge when DBS was turned on resulting in loss of 12 kg [32]. When DBS was switched off during the last four months of the study (for an improvement of sleep quality), nighttime binging returned and the patient regained the previously lost body weight [32]. These results might indicate the VMH as a promising target in the treatment of BED; however, further studies are needed to corroborate these preliminary results.

## 5. Potential Application of DBS in Anorexia Nervosa

In contrast to several pre-clinical studies in animal models for obesity and BED, the use of DBS in animal models of AN, including the most established model for AN, namely activity-based anorexia, is still missing. This animal model combines voluntary physical activity in a running wheel with time-restricted feeding resulting in a relevant body weight loss [68]. This lack clearly highlights the need for pre-clinical studies to better characterize the potential body weight-stimulating effects of DBS under conditions of experimentally induced anorexia.

Recently, a group focused on the SCC in the treatment of AN. The first study was published in 2013 and a follow-up study in 2017, both showing an increase of BMI in patients with refractory AN [33,41]. In the first study, DBS of the SCC in anorexic patients (bilateral, 130 Hz, 5–7 V) increased BMI in three out of six patients, who maintained their improved BMI after nine months [41]. The mean BMI of all six patients increased from 13.7 to 16.6 kg/m^2^ [41]. These results were confirmed and extended in the second study, showing an increase of mean BMI from 13.8 to 17.3 kg/m^2^ after 12 months following DBS of the SCC in 14 AN patients [33]. Depressiveness also improved as reflected by a decline in HAMD (Hamilton Depression Scale) and BDI (Beck Depression Inventory) scores, obsessive behavior improved as reflected by a decline in the Yale-Brown Obsessive Compulsive Scale score, scores for eating disorder rituals were reduced and the quality of life increased in three out of six patients at six months following surgery [41]. The improvements of disordered eating, obsessive behavior and depressiveness were confirmed in the follow-up study after twelve months using the same questionnaires. Also, anxiety was assessed and improved in four out of sixteen patients [33]. Two out of six patients did not have any serious side effects, whereas in four patients, adverse events occurred including pancreatitis, hypokalemia, re-feeding delirium, hypophosphatemia, worsening of mood and seizure in one patient [41]. The authors state that these adverse side effects were unrelated to the treatment and rather related to AN itself [41]. Besides that, other adverse effects related to the procedure were pain, nausea, air embolus and in one patient an intraoperative panic attack during drilling the skull [41]. In addition to the side effects reported in the first study, one patient had a surgical site infection and one patient needed the device to be explanted [33]. These data show the DBS of the SCC might be a suited and—for most patients—tolerated method to induce body weight gain in patients with severe AN. Nonetheless, further studies are needed, especially those using a control group receiving sham stimulation.

Areas of the reward circuitry are interesting targets in the treatment of AN. DBS of the NAcc shell (130 Hz, 100 µA) as part of the reward circuitry increases body weight gain in rats without affecting behavior (including feeding and drinking behavior, locomotion or grooming) [34]. Since other studies could not show any differences in body weight gain following DBS with a higher electric current (130 Hz, 500 µA) [35], stimulation parameters seem to greatly impact on the observed effects. Bilateral DBS of the NAcc (180 Hz, 6 V) induced a mean 65% increase of body weight in four patients with severe and refractory AN without inducing any side effects [64]. These results are further supported by an independent study showing an increase of BMI in eight anorexic patients following bilateral stimulation of the NAcc (100 Hz, 6–8 V) after twelve months from 13.3 to 20.1 kg/m^2^ [65]. In this study, no long-lasting adverse effects or postsurgical complications were reported (only fever and headaches were detected which disappeared three to five days after surgery [65]). Taken together, preliminary animal and human studies focusing on DBS of the NAcc (shell and core) give rise to a potential application of DBS in severe and refractory AN and should be corroborated in a larger follow-up randomized controlled trial.

In addition, several case reports focused on the role of DBS in the treatment of AN. A study reported an increase of BMI in a 56-year old anorexic woman from 14.4 to 19.1 kg/m^2^ after two years of bilateral stimulation of the subgenual cingulate cortex (130 Hz, 5 mA) without detecting side effects [62]. A subsequent study in a 52-year old underweight woman with anorexic symptoms confirmed these results, showing decreased concerns about food and weight gain accompanied by an increase of BMI from 18.5 to 19.6 kg/m^2^ following bilateral stimulation of the subgenual cingulate cortex (120 Hz, 5 V) without detection of side effects [63]. Recently, bilateral stimulation (130 Hz, 4.3 V) of the bed nucleus of the stria terminalis was reported to reduce the anxiety for food and eating in a 60-year old anorexic woman resulting in stable food intake without adverse effects [66]. However, the BMI did not change over 24 months of evaluation [66]. Further research, preclinical and clinical, is needed to show whether these brain areas hold true as potential target sites for DBS to improve AN symptoms.

The GPi is another interesting area tested for DBS in patients with PD. Despite the fact that the effects of DBS of the GPi are not as consistent as the results for the STN (one study showed a minimal, non-significant reduction in BMI following one year of stimulation [57], while another study showed a significant increase of BMI at four months after stimulation [43]), the application seems to be safe without severe side effects. Moreover, DBS of the GPi in patients with PD showed no adverse effect on verbal fluency [69], improved depression [70] and induces less neurocognitive impairments compared to DBS of the STN [71]. Regarding mood and cognition, there were no differences following unilateral DBS of the STN and GPi as shown in a randomized study with 45 subjects [72]. Therefore, the GPi might also be a promising brain target in the treatment of AN, especially in light of the finding that psychiatric complications seem to be less frequent and less severe in patients with PD following DBS of the GPi compared to the STN [73]. However, studies testing this hypothesis in AN patients are lacking so far. Further research directly focusing on body weight gain and psychiatric parameters following DBS of the GPi in patients with AN is therefore warranted.

Since DBS of the STN—a nucleus involved in motor functions but also reward processing—resulted in a reduction of nausea [58] and increased appetite [67] along with body weight gain [58,67] in patients with PD, targeting this area might also be of interest in patients with AN. Besides these positive effects, several side effects have been reported that should be kept in mind. The STN is involved in limbic functions and plays a central role in human emotions [74]. DBS of the STN has been reported to increase emotional lability [75] and in line with that, the suicide rate of patients undergoing DBS of the STN is 0.26% during the first year post surgery [76]. Whether the occurrence of suicide is due to DBS or subsequent to an alteration of depressiveness [77] needs further investigation. Moreover, the application of DBS of the STN has to be tested directly in patients with AN. Recently, it was shown that bilateral DBS of the STN (~140 Hz, 2.4 mA) increased the ‘wanting’ for low caloric foods (but not the ‘liking’) in patients with PD [44]. The desire for food is controlled by the brain reward system and can be divided in ‘wanting’ and ‘liking’. Interestingly, the dopamine striatal system is predominantly involved in ‘wanting’, whereas the opioid and cannabinoid systems are predominantly implicated in ‘liking’ [78]. Since dopamine is one of the main neurotransmitters in the reward circuitry [79], it is likely affected under these conditions. However, it is to note that these studies were performed in patients with PD and—although interesting—allow only limited transferability to AN patients and therefore, effects of DBS should be directly investigated in these patients. Lastly, due to the side effects reported the potential application of DBS in these areas should be carefully assessed and performed with great caution.

## 6. Challenges of DBS in the Treatment of Eating Disorders

Although current studies focusing on DBS in the treatment of eating disorders are promising and indicate a successful application, the cost-value ratio as well as ethic aspects must be discussed [80]. In PD, several studies have demonstrated the fiscal superiority of DBS over medical treatment. Indeed, the initial surgical cost are higher in the first year, but afterwards overall medication in patients with PD decreases and with that the expenses. For the treatment of obesity, DBS so far has higher surgical costs compared to bariatric surgery; however complication rates (at least the ones known so far) are lower. In consideration of the data for DBS in patients with PD, it is to say that DBS in the treatment of obesity might have higher initial costs but might decrease long-term costs. Especially for patients who failed bariatric surgery, DBS could be a promising alternative to lose body weight [80].

Besides that, there are also ethical considerations since DBS affects the neuronal network and with that the patients’ autonomy. Therefore, patients should be selected carefully and should already be refractory to medical or surgical treatment. The application of DBS should follow informed consent and stringent inclusion criteria in conducting clinical trials [80]. Therefore, under conditions of obesity DBS should only be considered in patients who failed drugs, psychological treatment and bariatric surgery, e.g., obviously due to persistent binge-eating behavior. The cost-value ratio of DBS in obesity will have to be evaluated in appropriate clinical trials with stringent inclusion criteria in refractory obese individuals. The same caution should be applied in AN, where DBS might become an option in patients with refractory AN that failed all other available treatment strategies. For AN, an ethical standard framework has been recently introduced that will help at reaching a cautious and well-balanced decision [81].

## 7. Conclusions

**Figure 1:**
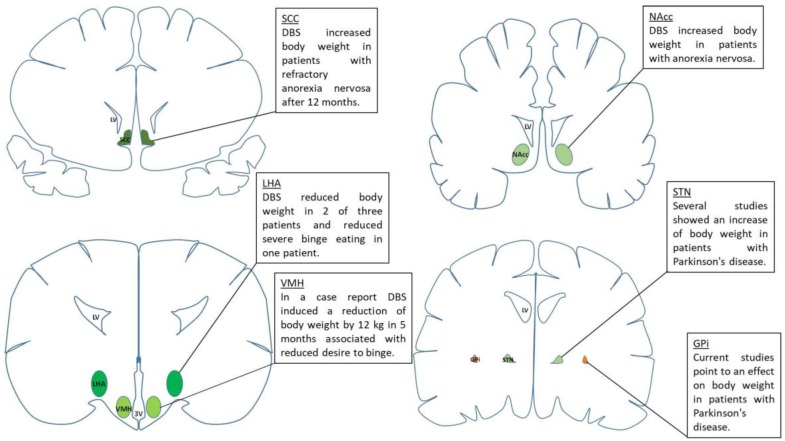
Schematic overview of human studies investigating the role of deep brain stimulation (DBS) in several brain areas to modulate food intake and body weight. The lateral and the ventromedial hypothalamus could be targeted in obese patients and stimulation might be useful to reduce the desire to binge. The nucleus accumbens, subcallosal cingulate and the subthalamic nucleus are potential brain areas that could be targeted in the treatment of anorexia nervosa. Abbreviations: 3 V, third brain ventricle; GPi, globus pallidus internus; LHA, lateral hypothalamus; LV, lateral brain ventricle; NAcc, nucleus accumbens, SCC, subcallosal cingulate; STN, subthalamic nucleus; VMH, ventromedial hypothalamus.

Although well established for patients with PD or depression, the application of DBS in obesity, BED and AN is still in its infancy and its broad use cannot be recommended yet. Despite the fact that various studies in animal models as well as in humans (Figure 1) showed promising results, the stimulation parameters used in DBS are far from being standardized, hampering comparability. Therefore, future studies are needed systematically and in a controlled fashion investigating the effects of DBS in the respective patient groups. Since for obesity and BED only animal studies or case reports are available, bigger clinical trials are urgently needed investigating respective patients. These studies will help to define target areas, stimulation parameters and to describe possible side effects.

## Figures and Tables

**Table 1:** Overview of current studies focusing on the effects of DBS on eating behavior and body weight.

Disease	Brain area	Mode	Species	Effect	Reference
**Obesity**	Nucleus accumbens shell	Unilateral, 160 Hz, 150 µA	Diet-induced obese mice	↓ food intake; ↓ body weight	[50]
	Nucleus accumbens shell	Unilateral, 130 Hz, 500 µA	Diet-induced obese rats	↓ food intake; ↓ body weight	[35]
	Nucleus accumbens	Bilateral, 185 Hz, 3.5 V	Obese patient	↓ body weight	[60]
	Nucleus accumbens	Bilateral, 130 Hz, 5 mA	Obese patient	↓ body weight	[61]
	Lateral hypothalamus	Bilateral, 180-200 Hz, 2 V	Diet-induced obese rats	- food intake; ↓ body weight	[53]
	Lateral hypothalamus	Bilateral, 130 Hz, 300 µA	Obese Zucker rats	↓ food intake; ↓ body weight	[37]
	Lateral hypothalamus	Bilateral, 185 Hz, 1–7 V	Obese patients	↓ body weight	[31]
	Ventromedial hypothalamus	Unilateral, 150/500 Hz, 10 µA	Sprague-Dawley rats	↓ body weight	[38]
	Ventromedial hypothalamus	Unilateral, 100 Hz	Dogs	↓ food intake	[55]
	Ventromedial hypothalamus	Bilateral, 50 Hz, 0.5–1.5 mA	Göttingen minipigs	↓ body weight	[39]
	Ventromedial hypothalamus	Unilateral, 80 Hz, 2V	Macaca fascicularis monkey	↓ meal size; body weight; ↓ body fat	[54]
	Ventromedial hypothalamus	Bilateral, 50 Hz	Obese patient	- food intake; ↓ body weight	[32]
	Central nucleus of amygdala	Bilateral, 20/130 Hz, 125 µA	Sprague-Dawley rats	↓ palatable food (sucrose)	[56]
	Rostromedial tegmental nucleus	Bilateral, 10 Hz, 20 µA	Sprague-Dawley rats	↓ food intake	[46]
**Binge-eating disorder**	Nucleus accumbens shell	Unilateral, 160 Hz, 150 µA	Mouse model of binge eating	↓ binge eating	[50]
	Nucleus accumbens core	Bilateral, 150 Hz, 150 µA	Rat model of binge eating	↓ binge eating	[52]
	Lateral hypothalamus	Bilateral, 185 Hz, 1–7 V	Obese patients	↓ severe binge eating	[31]
	Ventromedial hypothalamus	Bilateral, 50 Hz	Obese patient	↓ tendency to binge	[32]
**Anorexia nervosa**	Subcallosal cingulate	Bilateral, 130 Hz, 5–7 V	Anorexic patients	↑ body weight	[33,41]
	Subgenual cingulate cortex	Bilateral, 130 Hz, 5 mA	Anorexic patient	↑ body weight	[62]
	Subgenual cingulate cortex	Bilateral, 120 Hz, 5 V	Underweight patient	↑ body weight	[63]
	Nucleus accumbens	Bilateral, 180 Hz, 6 V	Anorexic patients	↑ body weight	[64]
	Nucleus accumbens	Bilateral, 100 Hz, 6–8 V	Anorexic patients	↑ body weight	[65]
	Bed nucleus of stria terminalis	Bilateral, 130 Hz, 4.3 V	Anorexic patient	- body weight, ↓ anxiety of food and eating	[66]
	Nucleus accumbens shell	Bilateral, 130 Hz, 100 µA	Sprague-Dawley rats	↑ food intake (first hour)	[36]
	Nucleus accumbens shell	Bilateral, 130 Hz, 100 µA	Sprague-Dawley rats	- food intake; ↑body weight	[34]
	Internal globus pallidus	Bilateral, 130 Hz, 2.6 V	Patients with PD	↑ body weight	[43]
	Ventromedial hypothalamus	Bilateral, 185 Hz, 2.5 -3.5 V	Vervet monkeys	↑ food intake	[40]
	Subthalamic nucleus	Bilateral, 240 Hz, 2.4 mA	Patients with PD	↑ high caloric and tasty food, ↑ body weight	[44]
	Subthalamic nucleus	Bilateral, 185 Hz, 2.9 V	Patients with PD	↑ body weight, ↑ appetite	[67]
	Subthalamic nucleus	Not mentioned	Patients with PD	↑ body weight	[57,58,59]

Abbreviations: ↑ increase; ↓ decrease; - no change; PD, Parkinson’s disease.
